# Impact of Genomics Platform and Statistical Filtering on Transcriptional Benchmark Doses (BMD) and Multiple Approaches for Selection of Chemical Point of Departure (PoD)

**DOI:** 10.1371/journal.pone.0136764

**Published:** 2015-08-27

**Authors:** A. Francina Webster, Nikolai Chepelev, Rémi Gagné, Byron Kuo, Leslie Recio, Andrew Williams, Carole L. Yauk

**Affiliations:** 1 Environmental Health Science and Research Bureau, Health Canada, Ottawa, ON, Canada; 2 Department of Biology, Carleton University, 1125 Colonel By Drive, Ottawa, Canada; 3 Integrated Laboratory Systems Inc., Research Triangle Park, North Carolina, United States of America; Sabanci University, TURKEY

## Abstract

Many regulatory agencies are exploring ways to integrate toxicogenomic data into their chemical risk assessments. The major challenge lies in determining how to distill the complex data produced by high-content, multi-dose gene expression studies into quantitative information. It has been proposed that benchmark dose (BMD) values derived from toxicogenomics data be used as point of departure (PoD) values in chemical risk assessments. However, there is limited information regarding which genomics platforms are most suitable and how to select appropriate PoD values. In this study, we compared BMD values modeled from RNA sequencing-, microarray-, and qPCR-derived gene expression data from a single study, and explored multiple approaches for selecting a single PoD from these data. The strategies evaluated include several that do not require prior mechanistic knowledge of the compound for selection of the PoD, thus providing approaches for assessing data-poor chemicals. We used RNA extracted from the livers of female mice exposed to non-carcinogenic (0, 2 mg/kg/day, mkd) and carcinogenic (4, 8 mkd) doses of furan for 21 days. We show that transcriptional BMD values were consistent across technologies and highly predictive of the two-year cancer bioassay-based PoD. We also demonstrate that filtering data based on statistically significant changes in gene expression prior to BMD modeling creates more conservative BMD values. Taken together, this case study on mice exposed to furan demonstrates that high-content toxicogenomics studies produce robust data for BMD modelling that are minimally affected by inter-technology variability and highly predictive of cancer-based PoD doses.

## Introduction

Toxicogenomics is expected to become an asset to human health risk assessment because it provides mechanistic data in a more efficient and cost-effective manner, using fewer experimental animals than the majority of standard toxicity testing methods. Toxicogenomics data can be used to determine the molecular mode of action (MoA) of chemical carcinogens and has been shown to be predictive of genotoxicity and cancer outcomes [1–13]. For these reasons, many regulatory agencies worldwide are exploring ways to incorporate toxicogenomic data into their chemical risk assessments. The major challenge in using toxicogenomics data for this purpose lies in determining how to distill these complex datasets into manageable pieces of usable information. Ultimately, if toxicogenomics data are to be included in chemical risk assessment, a consensus must be reached regarding which genomics platforms are appropriate, how the data should be modeled, and how to choose appropriate point of departure (PoD) values.

DNA microarrays have been the predominant technology used in toxicogenomics studies over the past decade. However, microarrays are experimentally constrained by pre-defined probe sequences and limited dynamic ranges. RNA-sequencing (RNA-seq), which does not have these experimental limitations, is beginning to be used more routinely to quantify transcript abundance. RNA-seq also provides information on alternative splicing, novel transcripts, and, since it is possible to adjust sequencing depth, can detect low abundance transcripts with greater accuracy than microarrays [14]. However, there are currently no established best practices for handling RNA-seq data (e.g., to align, filter, normalize, and identify differentially expressed RNAs), which represents a barrier to its use in applied and regulatory settings (as these require a degree of experimental reproducibility and transparency). In order to consider RNA-seq as an alternative to microarrays, it will be important to understand how these two high-content technologies compare with respect to the mechanistic insight and quantitative outputs they produce.

One important quantitative metric in risk assessment is the benchmark dose (BMD), which is the dose at which there is a change in a biological response compared to background levels. BMD modeling was adopted by the United States Environmental Protection Agency (EPA) to improve upon traditional NOAEL/LOAEL (no observed adverse effect level; lowest observed adverse effect level) approaches [15]. Since toxicogenomics studies produce a very large amount of data, most of which can be BMD modeled, these studies generate a huge number of BMD values (representing BMDs for individual genes, molecular pathways, gene ontologies, and more) from which a single PoD must be identified. A PoD is typically chosen as the dose at which an important, disease-predicting biological response departs from background levels. Overall, it is clear that a good understanding of the effects of the genomics platform and data processing strategies on the dose response curves of transcriptomic endpoints is essential.

Two recent studies have evaluated the relative abilities of RNA-seq and microarrays in producing comparable and reliable quantitative data for human health risk assessment. To determine whether a shift from DNA microarrays to RNA-seq might influence transcriptional BMDs, Black et al. [16] applied BMD modeling of genes and pathways for bromobenzene-dependent gene expression changes in rat liver measured using both RNA-seq on a 5500xl Series SOLiD Next Generation Sequencer and Affymetrix microarrays. These authors reported low to moderate concordance (r = 0.2–0.5) between the two technologies for pathway-based BMD values. The modest pathway-based BMD correlation was attributed to differences in the dynamic range of the two technologies and the different normalization methods used. Wang et al. [14] compared toxicant-dependent changes in rat liver gene expression between the two platforms for 27 chemicals. They reported greater inter-platform concordance of gene expression for chemicals that elicit larger transcriptional effects (both in magnitude of fold-change and in number of differentially expressed genes, DEGs). They also reported that the low inter-platform correlation between DEG fold change is attributable to the inferior ability of microarrays to detect low abundance transcripts. These two studies highlight some of the difficulties encountered when comparing microarray and RNA-seq data. Therefore, they demonstrate the need for additional research to address these inter-platform inconsistencies.

In the present study we compared the dose response of transcriptomic data obtained from samples using three genomics platforms: Agilent DNA microarrays, Illumina poly-A RNA-sequencing and custom RT^2^ Profiler PCR arrays. We used RNA extracted from the livers of female mice sub-chronically exposed to furan, a rodent liver carcinogen, to make inter-platform comparisons of DEG fold-change, gene BMDs, and pathway mean- and median-BMDs. We contrasted these transcriptomic BMDs directly against the published BMDs for furan-induced cancers. We further compared BMD values derived from full gene lists to those derived from gene lists that had been filtered for differential gene expression. Finally, we explored two established and two novel approaches for PoD selection for transcriptional data: 1) the lowest BMD(L)-mean for a molecular pathway [17,18]; 2) the lowest BMD(L)-mean for a molecular pathway that has been validated using a second gene expression platform; 3) the mode, mean, and median of all pathway BMD(L)-means; and, 4) the BMD of a key MoA-based signaling pathway [3,19]. The purpose of this study is to understand how inter-platform differences affect gene expression data, particularly the dose-response, in order to increase user confidence in toxicogenomics data and to propose best practices for use in chemical risk assessment.

## Materials and Methods

### Animals and exposures

Animal exposures have been described elsewhere [3]. Briefly 6–7 week old female B6C3F1 mice were housed five per cage in polycarbonate cages in a specific pathogen free (SPF) and Association for Assessment and Accreditation of Laboratory Animal Care (AAALAC) accredited facility. Mice were exposed by oral gavage to 0, 2, 4, or 8 mg/kg bodyweight per day (mkd) furan for 21 days (n = 5 per dose). Four hours after the final dosing mice were anesthetized by CO_2_ inhalation prior to euthanasia by exsanguination. Livers were removed and pieces were flash frozen and stored at or below -70°C. All procedures were conducted in compliance with the Animal Welfare Act Regulations (9CFR1–4) at Integrated Laboratory Systems, Inc. (ILS). ILS is an AAALAC accredited facility (AAALAC International File number: 000810). Mice were handled and treated according to the guidelines provided in the National Institutes of Health (NIH) Guide for the Care and Use of Laboratory Animals (ILAR, 1996; http://dels.nas.edu/ilar/). The animal use protocol for this study was approved by the ILS Institutional Animal Care and Use Committee (IACUC), Research Triangle Park, NC. ILS is an Office of Laboratory Animal Welfare (OLAW) registered, Class R Research Facility (OLAW assurance 3490–01).

### RNA extraction

RNA was extracted from frozen liver tissue using the RNeasy Midi RNA extraction kit (Qiagen, Mississauga, ON, Canada), quantified using a NanoDrop Spectrophotometer, (Thermo Fisher Scientific Inc., Wilmington, DE, USA), and qualified using a 2100 Bioanalyzer (Agilent Technologies, Mississauga, ON, Canada) as previously described [3].

### RNA-Sequencing (RNA-seq)

Standard polyA-enrichment Illumina RNA-seq libraries were prepared following Illumina's TruSeq v2 Library Preparation protocols. Briefly, polyA-enriched RNA from 4 biological replicates per dose group was chemically fragmented, primed using random hexamers, and converted into cDNA libraries. Illumina adapters were ligated to the resultant libraries and libraries were paired-end sequenced on a HiSeq2000 with a sequencing depth of 40 million reads and a read length of 100 base pairs. The data were processed using Illumina's Real Time Analysis software, and converted into FASTQ files using CASAVA software (Illumina). The FASTQ files were aligned to the GRCm38 mouse genome with STAR [20] using default parameters. Reads that did not align were discarded from any further analyses. Gene expression values were calculated for the Ensembl Gene Set (GRCm38v75). Feature counting was performed with Python using HTSeq-count (version 0.6.1) [21] with the m parameter set to “intersection-nonempty”. DEG analysis was then performed using the limma pipeline [22] for the following set of contrasts: control– 2 mkd furan, control– 4 mkd furan, control– 8 mkd furan. Genes were considered ‘present’ if there were at least 0.5 counts per million (cpm) in at least 3 of 4 samples, in at least one dose group (and were called ‘absent’ if they did not pass this filter). TMM normalization was applied before BMDExpress analysis. The dataset is publically available through the Gene Expression Omnibus (GEO, http://www.ncbi.nlm.nih.gov/geo/): GSE64371.

### Microarray

The microarray experimental design and analysis has been previously described [3,23] and the full dataset is available through GEO: GSE48644. Cy5-labeled sample cRNA were hybridized against a Cy3-labeled universal mouse reference cRNA (Stratagene by Agilent Technologies Inc., Mississauga, ON, Canada) on SurePrint G3 Mouse GE 8×60 K microarrays (Agilent Technologies Inc., Mississauga, ON, Canada). A randomized block design (in which the slide was treated as the blocking effect) was used. To obtain DEGs, median signal intensities were normalized using LOWESS [24] in R [25] and probes with technical replicates were averaged. Expression levels were determined using the microarray analysis of variance (MAANOVA) library [26]. Probes were given a ‘present’ call if the signal intensity was at least three standard deviations above the background non-murine control spots on the array (and absent if they were below this threshold). The Fs statistic [27], a shrinkage estimator, was used to determine gene-specific treatment effects, and the associated p values were estimated using the permutation method (30,000 permutations with residual shuffling) and were adjusted for multiple comparisons using the false discovery rate (FDR) approach [28]. Fold changes were estimated using least square means of each pairwise comparison. Genes having an FDR-adjusted p ≤ 0.05 and a fold change ≥ ±1.5 were considered differentially expressed. Upon removal of outliers (arrays with high background), the final sample sizes used for gene expression analysis were n = 5, 5, 4, 5 for 0, 2, 4, 8 mkd furan dose groups, respectively.

### PCR array

Custom RT^2^ Profiler PCR arrays were designed with 88 target genes, 5 housekeeping genes, and 3 internal controls (SA Biosciences by Qiagen, Frederick, MD, USA). The 88 target genes were chosen based on the results of microarray experiments. The majority of genes chosen were differentially expressed (up-regulated or down-regulated) by the highest dose of furan (8 mkd). The rest were chosen because they were also (or only) differentially expressed at lower doses (2 mkd: Saa1, Esr1, Cyp51, Slc10a2; 4 mkd: Ccng1, Pdgfa, Rnd2, Slc23a3, Bax, Aen, Tubb2c, Cdkn1a, Tuba1c, Ces2, Gdf15). 500 ng samples of RNA (n = 5 samples per dose group) were reverse transcribed using RT^2^ First Strand kits (Qiagen). cDNA was combined with RT^2^ SYBR Green Mastermix (Qiagen) and was thermocycled according to: 1x (10 min at 95°C); 40x (15 seconds at 95°C, 1 minute at 60°C), on a CFX96 Thermocycler (BioRad Laboratories, Mississauga, ON, Canada). Fold change values were calculated using the deltaCt method using the RT^2^ Profiler PCR Array Data Analysis web tool, version 3.5 (http://pcrdataanalysis.sabiosciences.com/pcr/arrayanalysis.php). Normalized deltaCt values were used for dose response modeling in BMDExpress.

### BMD modeling

BMDExpress version 1.4.1 [29] was used to perform BMD analysis on RNAseq, microarray, and PCR array datasets. Only genes for which there was a ‘present’ call in at least one dose-group (i.e., 4 out of 5 samples with ‘present’ call for that gene in at least one dose group for microarrays; or, 3 out of 4 samples with > 0.5 cpm for the gene in at least one dose group for RNA-seq) were modeled. Prior to modeling in BMDExpress, these datasets were then pre-filtered for differential gene expression in three ways: (1) FDR p < 0.05 (in at least one dose); (2) ANOVA p < 0.05 (in at least one dose); (3) no pre-filtering. The three filtering conditions were not applicable to the qPCR data because all genes measured by qPCR were significantly changed in at least one dose. Constant variance was assumed for the RNA-seq data in order to analyze it using the models that are currently available in BMDExpress. While the models in BMDExpress were not originally intended for count-based datasets, it has been used previously [16] and it was necessary to use the same software for the analysis of data from all platforms in order to make cross-platform comparisons. Hill, Power, Linear and Polynomial (2° and 3°) models were used to model gene expression dose-response. For each gene, the best fitting model was selected based on: (1) a nested chi-square test (cut-off of 0.05) that was used to choose between linear and polynomial models, followed by (2) the lowest Akaike Information Criterion (AIC) value for the nested, Hill and power models, and (3) a curve goodness of fit p > 0.1. Genes with BMD values that were higher than the highest dose were excluded. Model parameters included: maximum iterations (the convergence criteria for the model) set to 250, confidence level (the statistical lower confidence limit applied to the model that is used to determine the BMDL) set to 0.95, benchmark response (BMR, which is equivalent to the number of standard deviations defining the BMD) set at 1.349 (which corresponds the amount of change required to shift the mean response by10% above background), and a restriction of the power parameter to ≥ 1. The Hill model was restricted and flagged if the k parameter of the model was less than one third of the lowest positive dose, as per Black et al. [30]. In the case of a flagged Hill model, the next best model was selected only if it had a goodness of fit p > 0.05. In the case when no other model had p > 0.05, the Hill model was used and modified to 0.5 of the lowest BMD value. The resulting datasets were mapped to Ingenuity Pathway Analysis (IPA) canonical pathways, which were downloaded on April 24, 2014. Mean and median BMD(L)s were reported for pathways with at least three DEGs; however, PoDs were derived from pathways with a stricter threshold of at least 4 DEGs.

BMD software (BMDS250) was used to model the cancer dose response for hepatocellular adenoma (HCA) and carcinoma (HCC) from Moser et al. [31] and the dose response of the number of DEGs at each dose from each genomics platform. Methods for the former were reported previously in Jackson et al. [3]. For the latter, the BMDS wizard v1.9 was used (http://www.epa.gov/ncea/bmds/). Data were treated as dichotomous and the number of DEGs in any one dose was modeled as a percentage of the total number of DEGs summed across all doses. The benchmark response (BMR) was set to 10% extra risk as recommended in the Benchmark Dose Technical Guidance document [32]. BMD(L) values were chosen based on the lowest AIC value of models with goodness-of-fit p > 0.1 and BMD values (across all models) that differed by less than 3-fold.

## Results

### Inter-technology comparison of differentially expressed genes

**Fig 1:**
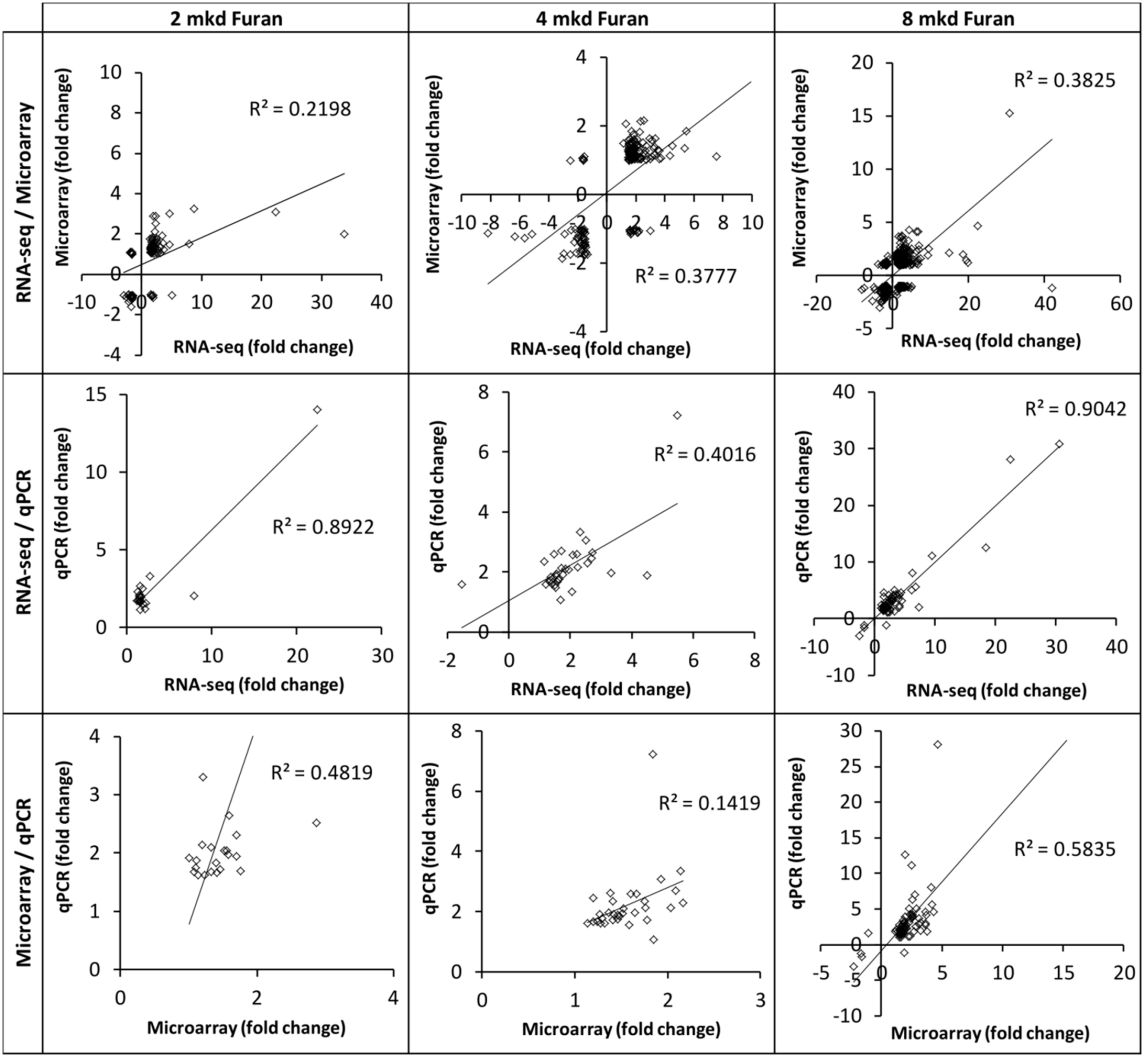
Gene expression levels are correlated between genomics platforms. Correlation analysis of the fold changes of differentially expressed genes (FDR p < 0.05, fold change > ±1.5 in at least one dataset) for 2 mkd (left), 4 mkd (middle), and 8 mkd (right) furan relative to controls. All regressions were significant (p < 0.05).

There was a dose-dependent increase in the number of DEGs on each platform. The number of DEGs obtained for each dose group on each platform, together with the BMD of their dose response, is summarized in Table 1 (S1–S4 Tables). The magnitude and direction of fold changes for the DEGs were compared between technologies (Fig 1). The magnitudes of fold changes produced by RNA-seq and qPCR were generally greater than those produced by microarrays, which is consistent with signal compression produced by two-color microarrays. All comparisons yielded significant correlations (linear regression p < 0.05). The RNA-seq/microarray correlations were the weakest (R^2^ = 0.220–0.382), in agreement with previous report of Black and co-workers [16]. Interestingly, the RNA-seq/qPCR correlation was much stronger (R^2^ = 0.401–0.904 vs 0.142–0.583), despite the fact that microarray data were used to design PCR arrays. For each inter-technology comparison, the best correlations were produced at the highest dose. Consistent with previous reports [14,16], the overlap of DEGs between technologies improved when the treatment effect was large (S1 Fig). Overlap of enriched pathways at the high dose approached 50% (S1 Fig).

**Table 1:** Gene and cancer dose response. The number of differentially expressed genes (by unique gene symbol) and their dose response (upper), and the BMDs of furan-dependent liver cancers (lower).

	Number of DEGs per dose group		
	RNA-seq	Microarray	qPCR
**2 mkd**	131 (12%)	17 (5%)	23 (26%)
**4 mkd**	221 (20%)	28 (8%)	32 (36%)
**8 mkd**	1041 (94%)	350 (98%)	71 (81%)
**Total DEGs** ^†^	1113	356	88
**BMD(L)** ^‡^	**1.90 (0.53)**	**2.78 (1.04)**	**0.67 (0.37)**
		**Cancer BMD values** *	
		*Model*	**BMD(L)**
Hepatocellular adenoma (HCA)		*Multistage*	**2.6 (1.6)**
Hepatocellular carcinoma (HCC)		*Logistic*	**5.1 (4.2)**
HCA or HCC		*Multistage*	**2.3 (1.3)**

DEGs are defined as a gene with an FDR p < 0.05, fold change > ±1.5

^†^Total number of unique DEGs across all three dose groups (within each platform)

^‡^Modeled in BMDS; dichotomous data presented as percent present; BMR = 10% extra risk

*Data from Moser et al. 2009

### Effect of pre-filtering gene expression data prior to BMD modeling

There is some debate regarding whether or not high-content gene expression datasets should be pre-filtered (to remove genes that do not respond to the treatment in a statistically significant manner in at least one dose), and the extent to which they should be filtered (i.e., whether there should be an adjustment for multiple testing), prior to modeling in BMDExpress. To address this issue, we performed BMD modeling for the RNA-seq and microarray datasets using three different approaches. BMD modeling was applied to: (1) unfiltered gene expression data, (2) low-stringency pre-filtered data (requiring a gene to achieve an ANOVA p < 0.05 in at least one dose relative to control), or (3) high-stringency pre-filtered data (requiring a gene to achieve FDR p < 0.05 in at least one dose compared to control).

**Fig 2:**
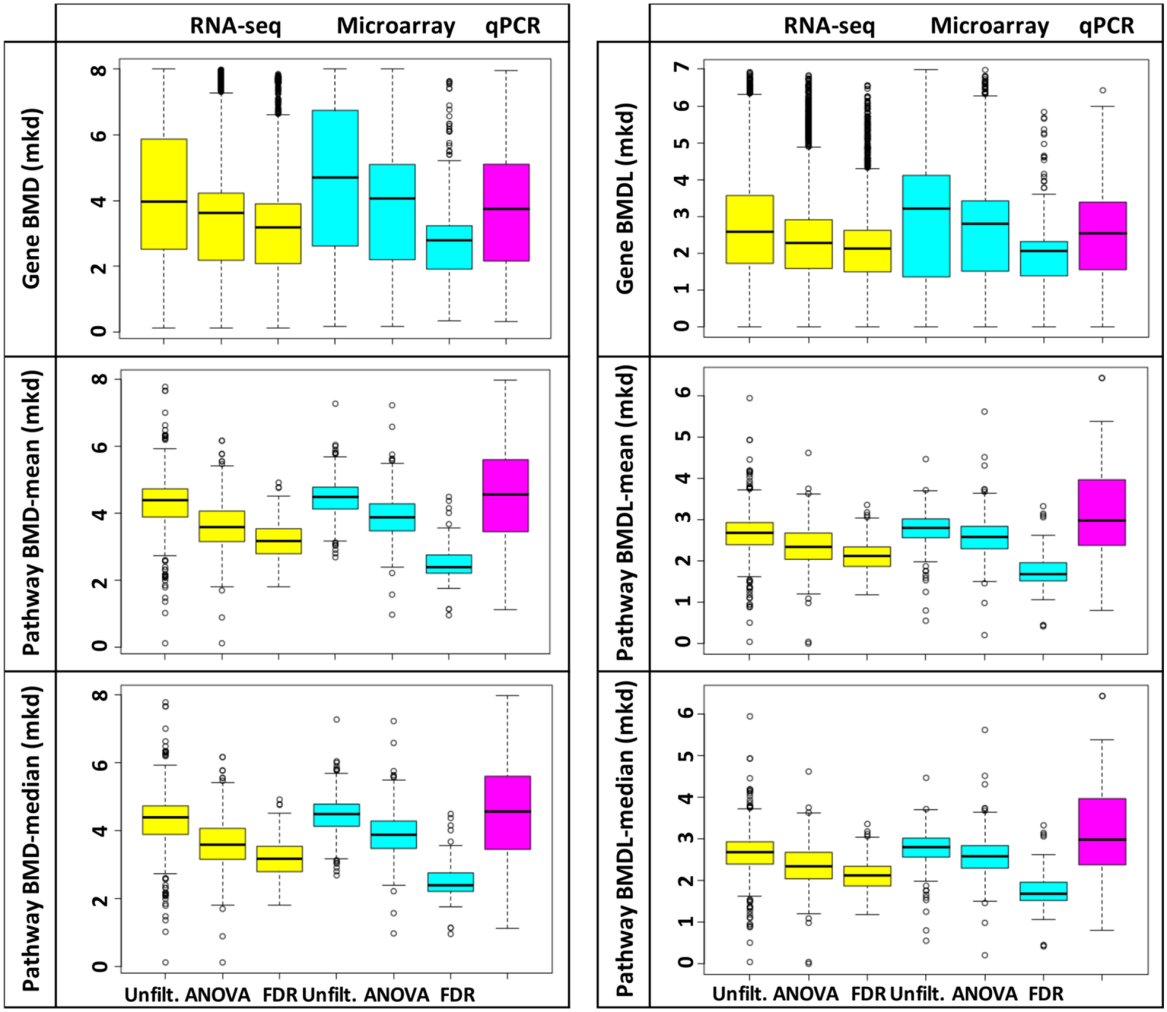
Filtering gene expression data significantly changes mean values for gene and pathway BMD(L)s. The distributions of the BMD (left) and BMDL (right) values for genes (top), pathway means (center), and pathway medians (bottom), from the RNA-seq (yellow), microarray (aqua), and qPCR (pink) experiments, were compared. Upper and lower quartiles are indicated by box, the median is indicated by the line within the box and points represent outlier BMD(L) values (upper quartile + 1.5 x the inter quartile range (IQR) or the lower quartile—1.5 x IQR). Within each genomics platform, filtering significantly changed sample means (t-test p < 0.0001; S5 Table).

**Fig 3:**
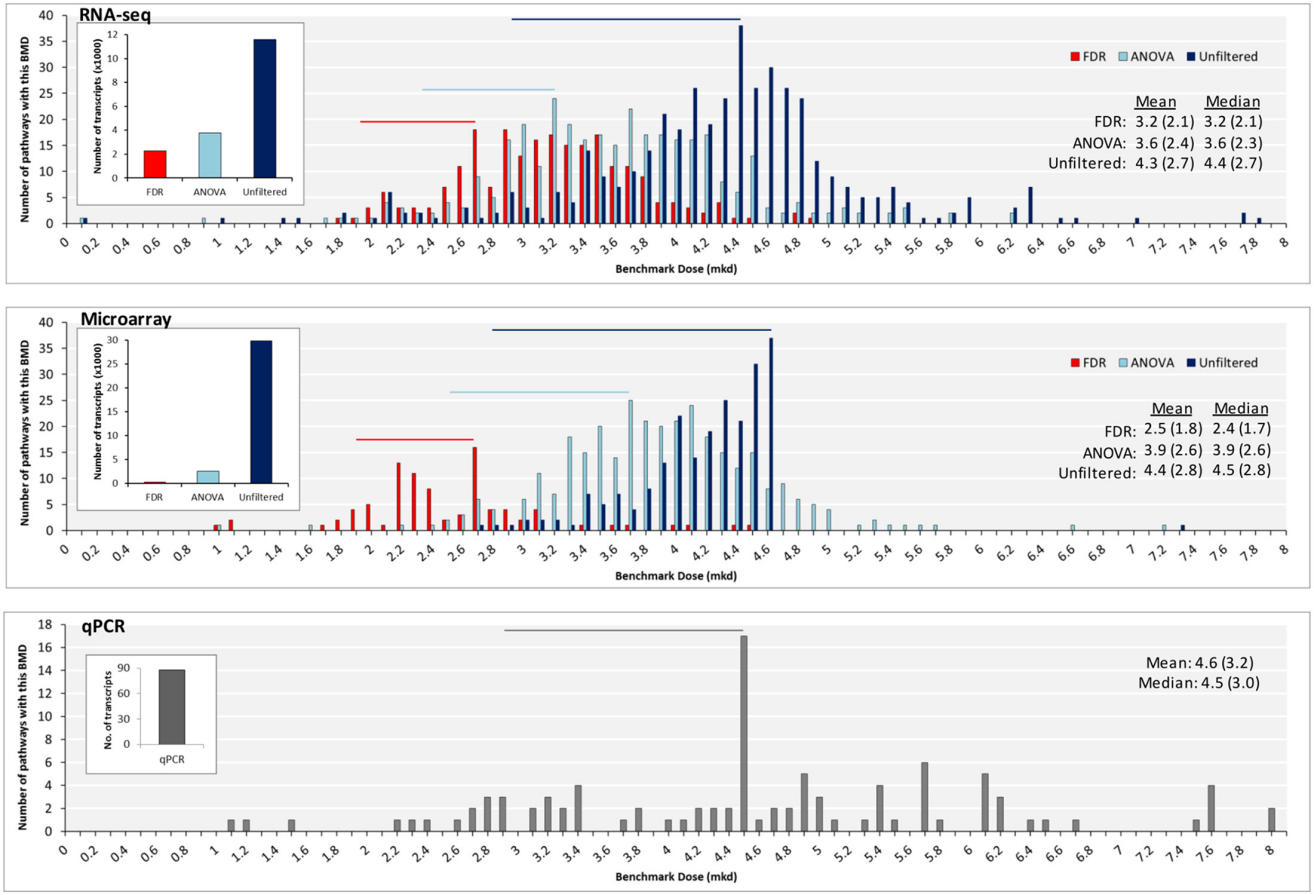
Filtering gene expression data changes the distribution of pathway BMD-mean values. Distributions of pathway BMD-mean values for RNA-seq (top), microarray (center) and qPCR (bottom). Mode BMD (BMDL) values are labeled. Modes, means and medians decrease as filtering stringency increases. Pathways were only considered in this analysis if they had 4 or more molecules with p fit>0.1. Overlain bar charts indicate the number of transcripts used to model each group.

The number of transcripts modeled for each approach produced by the RNA-seq platform was: (1) 11599 for unfiltered, (2) 3778 for ANOVA p < 0.05, (3) 2295 for FDR p < 0.05. The number of transcripts modeled for each approach produced by microarrays was (1) 29847 for unfiltered, (2) 2597 for ANOVA p < 0.05, (3) 364 for FDR p < 0.05. Applying a low- or high-stringency filter for statistically significant DEGs prior to modeling in BMDExpress significantly decreased the means of each group of BMD(L) values for both RNA-seq and microarray datasets, as shown in Fig 2; the results of the t-tests were extremely significant, with p-values being less than 0.0001 for all comparisons (S5 Table). Data were unimodally distributed. Mode, mean and median values were consistent across platforms (shown for pathway BMD-means, Fig 3, and pathway BMDL-means, S2 Fig), but were affected by filtering. The variety of the models used (Hill, Power, Linear, Polynomial 2° or 3°) to produce gene BMD(L)s was dictated by the genomics platform (as opposed to filtering for DEGs; S3 Fig); data filtering had the effect of streamlining BMD/BMDL plots (S3 Fig). After considering these data, the EPA’s criteria for modeling in BMDS, which stipulates that ‘there should be at least one statistically or biologically significant dose-related trend in the selected endpoint’ [32], and the risk assessors perspective (which is typically to use the most conservative estimates), we recommend that statistically filtered data be used. However, for the sake of comparison, we have included the results of most analyses for all three approaches.

### Inter-technology comparison of BMD values

**Fig 4:**
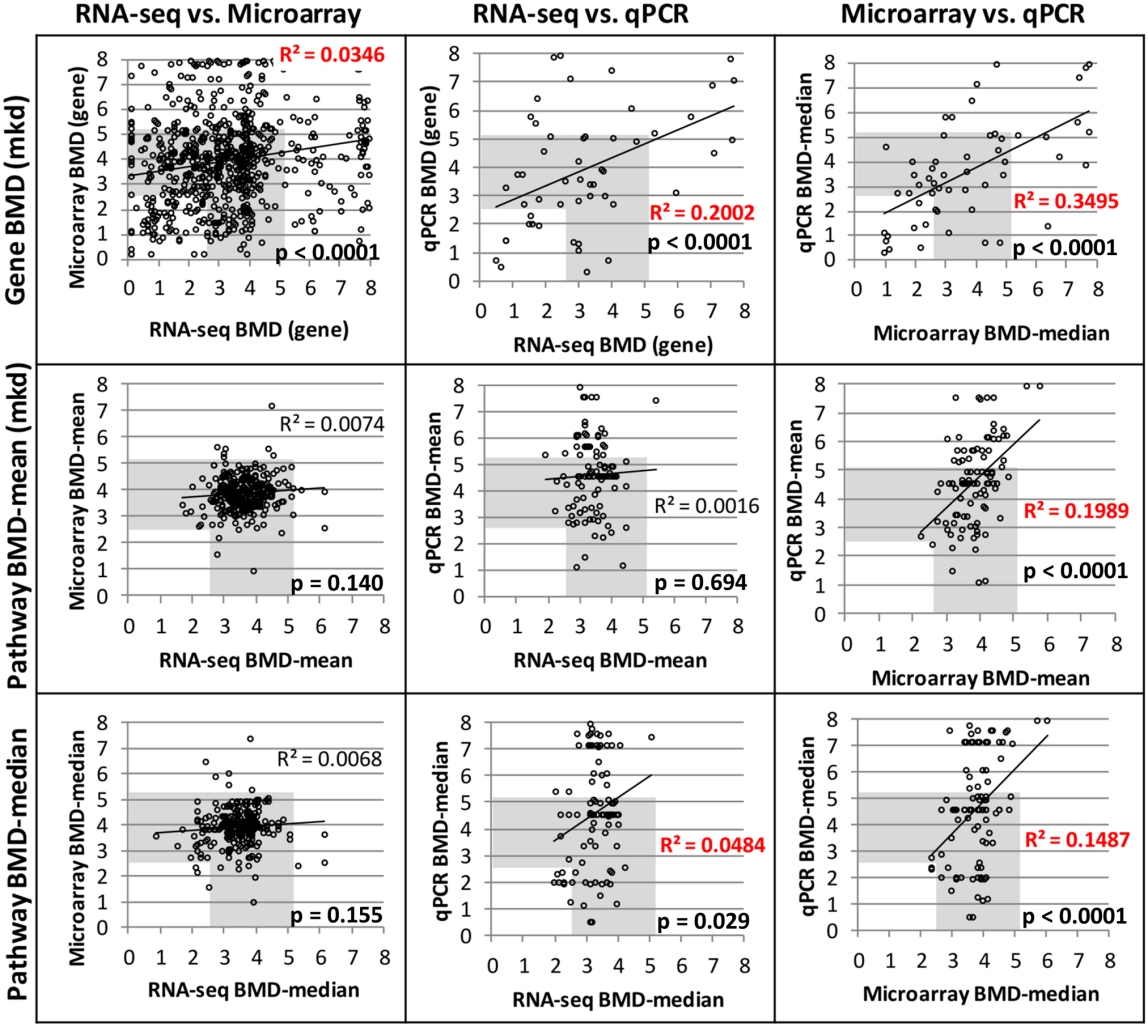
BMD values are not well correlated between genomics platforms. Inter-platform comparisons of BMDs for genes (top), pathway means (center), and pathway medians (bottom) for ANOVA filtered data. The BMD range for furan-induced HCA-HCC of 2.6–5.1 mkd is shaded in grey. Statistically significant correlations are indicated in red (regression p < 0.05).

The inter-technology relationships between BMD (or BMDL) values for genes or pathways were weak (R^2^ = 0.007–0.35) regardless of level of filtering (Fig 4; S4–S6 Figs). This poor correlation is consistent with the weak inter-technology correlation for the fold changes, noticeable from Fig 1. However, we observed that the cloud of data points was always concentrated in the furan-dependent liver cancer ‘PoD range’, which is defined here as the range between the HCA BMD (2.6 mkd) and the HCC BMD (5.13 mkd). This trend is especially clear for the RNA-seq versus microarray plots and the ANOVA- or FDR-filtered data. Quantitatively, analysis of the ANOVA-filtered RNA-seq, microarray, and PCR data revealed that 54.9, 47.6, and 49.3% of the gene BMDs, and 90.4, 94.1, and 62.6% of the mean pathway BMDs were within the bounds of the HCA-HCC BMDs, respectively (S6 Table). Therefore, while there is no clear inter-platform relationship, the values are informative because they clustered closely together in the cancer PoD range.

**Fig 5:**
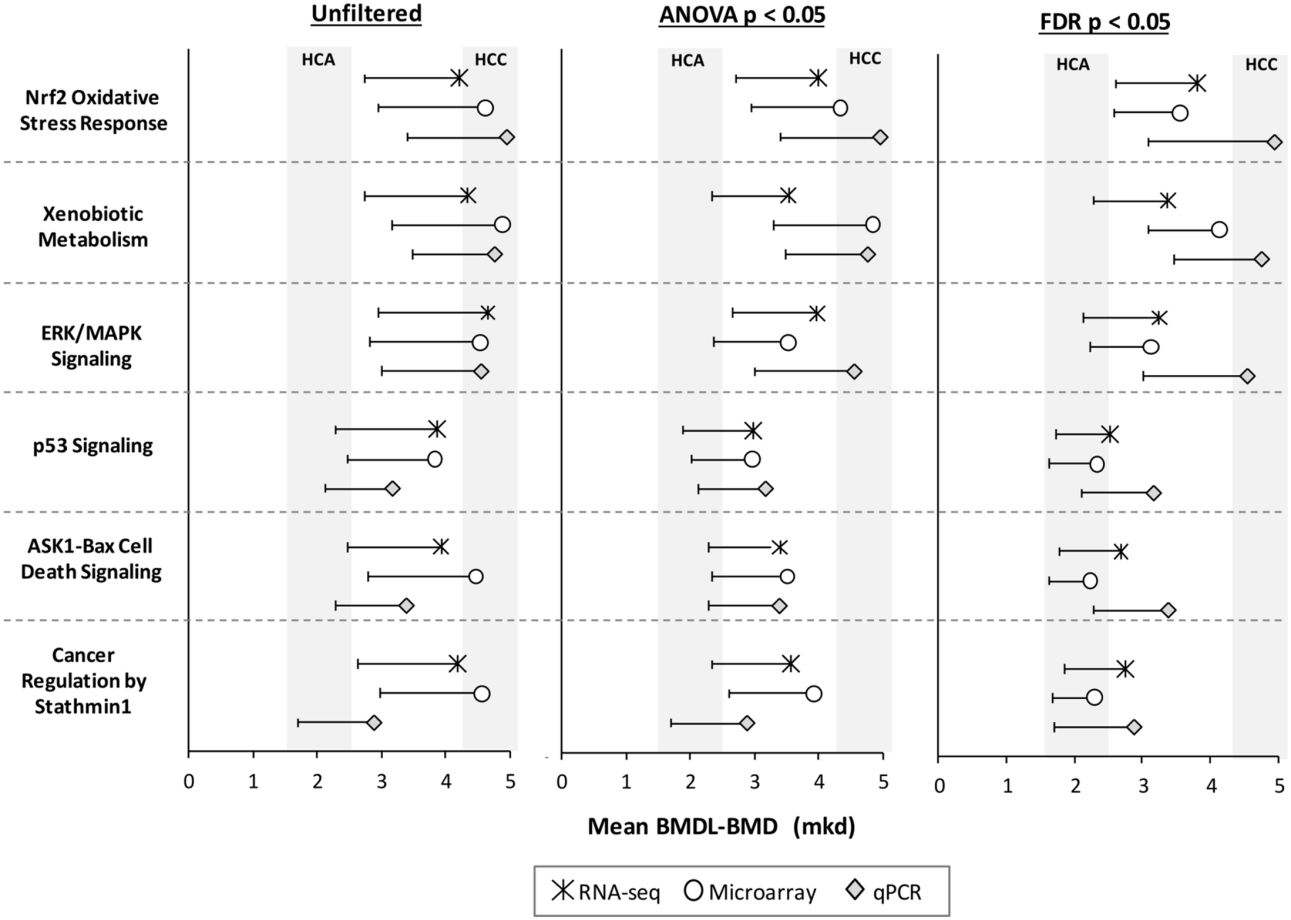
BMD-means of furan MoA pathways are consistent across platforms. Symbols indicate BMD values and whiskers indicate the lower confidence interval of the BMD. The confidence intervals (or the range between the BMDL and BMD) for HCA and HCC are shaded in grey.

We compared mean BMD values for molecular pathways that are important for furan’s carcinogenic MoA [3]: *Nrf2 Oxidative Stress Response*, *Xenobiotic Metabolism Signaling*, *ERK/MAPK Signaling*, *p53 Signaling*, *ASK1-Bax Cell Death Signaling* (renamed from: 14-3-3-mediated Signaling), and *Cancer Regulation by Stathmin1* (renamed from: Breast Cancer Signaling by Stathmin1). Pathway BMD-means were similar across technologies, with overlapping confidence intervals (Fig 5). Moreover, they were generally within confidence intervals of furan-induced HCA (BMDL-BMD, 1.6–2.6 mkd) or HCC (BMDL-BMD, 4.2–5.1 mkd). Importantly, not filtering the data prior to modeling pathways in BMDExpress generally produced higher BMD values with broader BMDL-BMD confidence intervals.

### Approaches for deriving transcriptional Point of Departure (PoD) values

**Fig 6:**
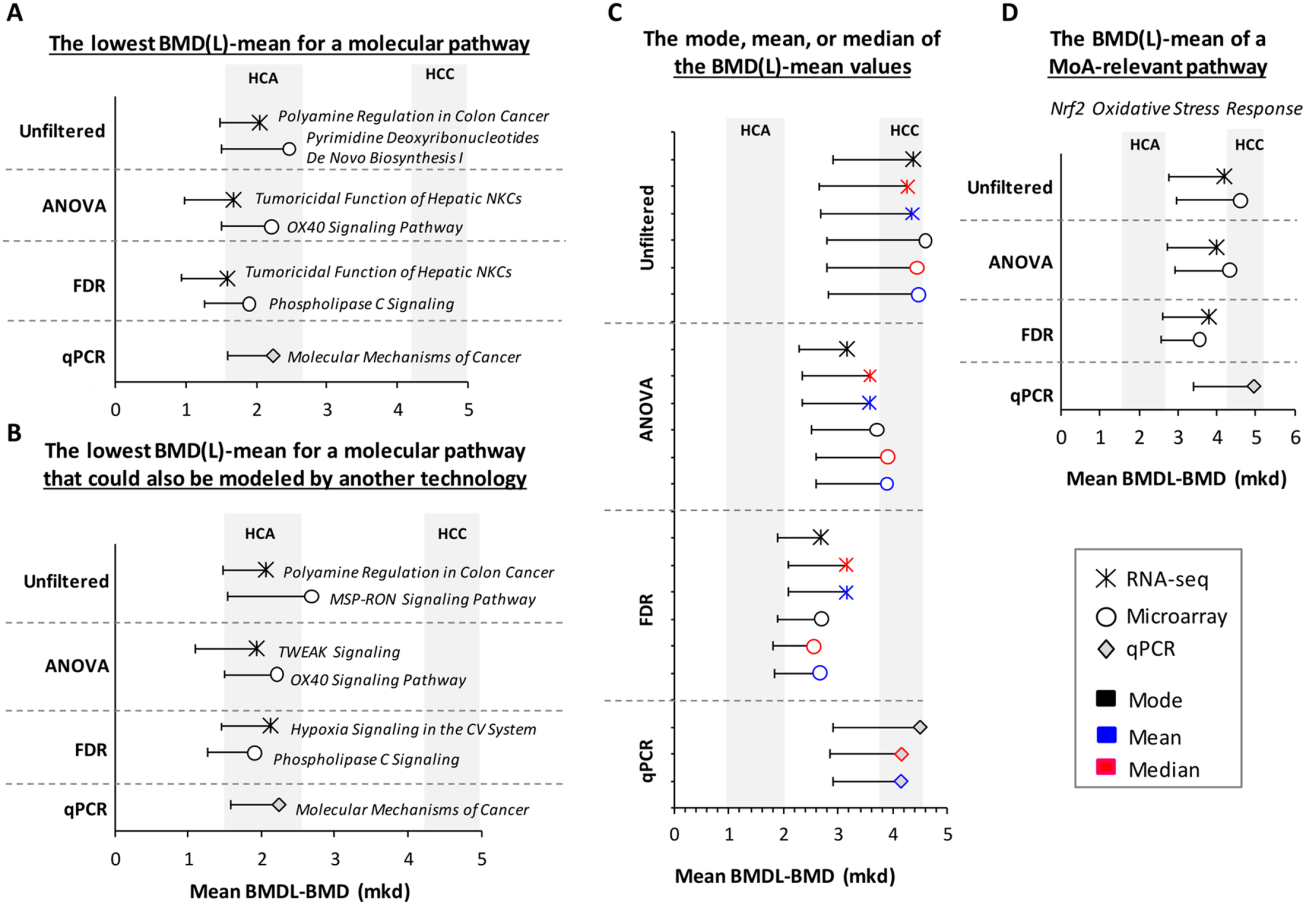
Four approaches to deriving transcriptomic PoD values. (A) the lowest BMD(L)-mean for a molecular pathway; (B) the lowest BMD(L)-mean for a molecular pathway that has been validated using a second gene expression platform; (C) the mode, mean, or median of the BMD(L)-mean values; and (D) the BMD of a key MoA-based signaling pathway. PoDs are represented as the pathway BMD-mean values (mkd) with lower confidence intervals indicated. All pathways had a minimum of four molecules that were modeled. The BMD confidence interval for each furan-dependent liver cancer is shaded in grey. NKC = Natural Killer Cells; CV = Cardiovascular; TWEAK = TNF-related weak inducer of apoptosis.

We considered four approaches for choosing a single PoD from the long list of transcriptional BMD values: (1) the lowest BMD(L)-mean for a molecular pathway; (2) the lowest BMD(L)-mean for a molecular pathway that has been validated using a second gene expression platform; (3) the mode, mean, or median of the BMD(L)-mean values; and (4) the BMD of a key MoA-based signaling pathway (*Nrf2 Oxidative Stress Response* pathway, in this case). The PoDs for each technology for these four approaches are summarized in Fig 6. The BMD values increased from approach 1–4. The first three approaches best approximated the HCA BMD (2.6 mkd), whereas the fourth, MoA-dependent approach best approximated the malignant form of furan-induced cancer, HCC (BMD: 5.1 mkd). Although the RNA-seq and microarray experiments relied on a much larger pool of DEGs, the qPCR-produced BMD-mean values were remarkably similar. Applying a statistical filter (ANOVA or FDR) changed the identity of the most sensitive pathway; however, the resulting BMD values that were generated by approaches 1 and 2 were quite similar in the filtered and unfiltered data. When looking at the distribution of the data (Fig 3), it is clear that mode, mean, and median values of the BMD-mean were affected by statistical filtering; however, within each group, they were quite consistent across technologies. The BMD-mean values produced for the *Nrf2 Oxidative Stress Response* pathway were quite similar between the filtered and unfiltered datasets and across technologies. Taken together, these four approaches to PoD derivation produced highly comparable results across platforms. Filtering the data tended to reduce the BMD value; however, the confidence intervals of the filtered and unfiltered datasets often overlapped.

## Discussion

In the present study we compared gene expression data obtained from furan-exposed mouse liver samples that were analyzed using three genomics platforms: Agilent DNA microarrays, Illumina polyA-enichment RNA-seq, and custom RT^2^ Profiler PCR arrays. We examined: 1) inter-platform consistency of DEG fold change, and DEG and pathway BMD values, 2) the effect of filtering data based on statistically significant changes in gene expression prior to modeling in BMDExpress, 3) the ability to produce transcriptional BMD values that are predictive of known cancer BMD values, and 4) different approaches for selecting a single PoD from a toxicogenomics dataset for use in risk assessment. Our findings provide insight into how toxicogenomics data from RNA-seq and microarrays align. This is critical for the transition to RNA-seq in toxicology, as the existing literature on the utility of toxicogenomics in risk assessment is predominantly derived from microarrays. Importantly, we demonstrate that important furan-dependent transcriptional changes occur in the liver at the same dose range as furan-dependent hepatocarcinogenesis regardless of technology. Therefore, our findings support the notion that transcriptional changes following sub-chronic treatments in rodents are prognostic of the harmful dose ranges at which adverse phenotypes occur.

Typically, when new technologies emerge they are compared against existing technologies. In genomics, qPCR is often used to validate microarrays, and now microarrays are being used to corroborate RNA-seq. However, DEG lists obtained using these two technologies often have modest overlap [14,16]. Overlap is known to improve when the degree of transcriptional perturbation is increased (i.e., at higher doses), which is what we observed for furan. The direction and magnitude of change of the DEGs that did overlap was usually consistent, and inter-technology DEG lists were all positively and significantly correlated (Fig 1). It is generally accepted that as the cost of next-generation sequencing continues to fall and the technical challenges inherent to processing, analyzing and storing these large datasets are overcome, RNA-seq is likely to become the dominant technology [33]. In fact, when comparing RNA-seq to microarrays some investigators have emphasized the inter-platform differences (as opposed to similarities) to make this point. For example, Zhao et al. [34] demonstrated the richness of RNA-seq data (which can measure novel transcripts and splice variants) over microarrays, and a greater ability to quantify low- and highly-expressed transcripts (which is more difficult on microarrays due to the limited dynamic range). However, while such studies are excellent for showcasing the technological superiority of RNA-seq, the low overlap of DEGs remains troubling since we do ultimately need to evaluate whether each technology is able to detect equivalent biological effects. Reassuringly, it appears that the inter-technology differences are less pronounced in higher-order biology (such as molecular pathways and gene ontologies). Overall, our results and the results of others [14] suggest that these two genomics platforms are most comparable when there is a large treatment effect and higher-order endpoints are analyzed.

In addition to comparing the biology detected by each platform, we were also interested to know how the transcriptional dose responses compared. Each platform showed a dose-dependent increase in number of DEGs and the BMDs of these increases were quite near the already known BMD for furan-induced HCA (Table 1). Producing BMD values for each DEG list was less straightforward; BMDExpress is flexible software that provides different options for filtering a dataset (based on statistically significant changes in gene expression) prior to modeling. However, since there is little guidance regarding the ‘correct’ approach for treating the data, the field has become divided as to what the ‘best practices’ should be. One argument is that no threshold for statistical significance of gene expression should be applied and that all genes that are detected should be modeled. This argument is based on the assumption that the greater the number of endpoints that are modeled, the more robust the BMDs will be. In contrast, others argue that only DEGs should be modeled, since these are the endpoints that actually responded to the chemical treatment. For the latter, various thresholds for differential expression are possible in the BMDExpress software, including an FDR p < 0.05 or a less stringent ANOVA p < 0.05. Indeed, it has been our experience that discrepant recommendations relating to the need (or lack thereof) to pre-filter data are made, which was one motivation for conducting this work. To our knowledge, we are the first to thoroughly investigate whether the application of these statistical filters has an effect on gene and pathway BMD values, which is fundamental to making evidence-based decisions regarding their use.

We observed that applying a filter for differential gene expression prior to modeling in BMDExpress significantly reduces the means of the gene and pathway (mean and median) BMD(L) values (Figs 2–3), indicating that more conservative BMD values are produced when only significantly changing transcripts are modeled. Importantly, the more stringent the filter, the better the mean and median BMD values approximated the BMD for furan-dependent HCA induction, which is not surprising given that applying these filters ensures that only the endpoints that truly respond to the treatment are considered. For this reason, we recommend that at least an ANOVA filter be applied to the data prior to modeling in BMDExpress. Indeed, for furan, after applying an ANOVA filter substantial pools of 3778 (RNA-seq) and 2597 (microarray) genes remain, which should allay concerns regarding the sufficiency of the number of endpoints modeled. Therefore, we contend that (at least in the case of furan) the argument that modeling a larger number of genes will produce a more exact BMD is erroneous.

As a whole, individual BMD values did not correlate well between platforms, regardless of level of filtering (Fig 4). This may be due to poor correlation of the fold changes seen in Fig 1 and reported previously [16] and is similar to the poor LOESS/KDMM correlation (r = 0.388) for BMD values of individual genes [16]. However, it is interesting to note that most of the transcriptional BMDs fell within the HCA-HCC BMD range and that precision increased filter stringency (S4–S6 Figs). Black et al. [16] also reported low to moderate concordance (r = 0.19–0.43) between RNA-seq and microarray for pathway BMD values in a study that compared the transcriptional dose-response in rat liver following exposure to bromobenzene, a hepatotoxicant. This notwithstanding, it has been well demonstrated that transcriptional and apical BMDs are often well correlated [1,3,18,19,35]. Thus, while there is a low inter-platform concordance of BMD values, they tend to cluster in a range that is prognostic of the doses at which adverse apical outcomes occur.

While it is encouraging that many of the gene and pathway BMDs cluster together in the same dose range that causes furan-induced liver cancer, it is still necessary to determine which BMD should be used as the PoD for risk assessment. We explored four approaches for POD selection using our furan data (Fig 6A). Using the first approach, the lowest mean BMD(L) for a molecular pathway (with at least four molecules), we obtained transcriptional BMD values for each genomics platform that ranged from 1.7–2.2 mkd, which are very close to the HCA BMD (2.6 mkd). In addition, the most sensitive pathways reported here for furan involve cell death-, Tumor Necrosis Factor (TNF)-, and cancer-related processes, all of which are relevant to furan’s MoA. These data support the idea that the lowest pathway BMD can be used as a surrogate PoD in the absence of apical data and that it is similar to the values established using traditional approaches. This approach has been used previously. For example, using five test chemicals, Thomas et al. demonstrated that the BMD and BMDL values of the most sensitive GO categories (measured following a 13 week chemical exposure) were predictive of PoD doses for cancer and non-cancer endpoints. They also showed that these transcriptional endpoints were well correlated with corresponding apical endpoints [17,35]. A recent case study of the genotoxic carcinogen benzo[a]pyrene also report similar transcriptional and apical BMDs for corresponding endpoints using the most sensitive transcriptional endpoints [1]. This approach of using the lowest BMD(L)-mean of a perturbed molecular pathway is useful for data-poor compounds (i.e., chemicals for which there are few, or no, apical data) because no knowledge of the MoA is required for PoD selection.

Our second proposed approach to PoD selection, to use the lowest mean BMD(L) for a molecular pathway that could also be modeled by another technology, is a safeguarded version of the first. We propose adding this validation step to the first approach in order to boost confidence in the PoD dose and avoid basing conclusions on one single assay or endpoint. In the case of furan, the most sensitive pathways for the microarray (*OX40 Signaling Pathway*) and qPCR (*Molecular Mechanisms of Cancer*) experiments remained unchanged; however, the most sensitive pathway for the RNA-seq experiment, *Tumoricidal Function of Hepatic Natural Killer Cells*, could not be modeled by either microarray or qPCR; thus, this pathway did not meet the criteria. The most sensitive RNA-seq pathway that could be modeled using data from at least one of the other two technologies was *TWEAK Signaling*. In this case, choosing a pathway that could be modeled using data generated from at least one other technology increased the PoD from 1.7 to 1.9 mkd (Fig 6B), which is slightly closer to the HCA BMD (2.6 mkd). These first two approaches to PoD selection each produce biologically plausible PoDs for furan. It appears that choosing a PoD based on one technology is sufficient; however, if there are data available from an additional platform, modeling and considering these data is worthwhile. Finally, we note that filtering for differential gene expression only marginally affected the values obtained using both ‘lowest BMD(L)’ approaches, suggesting that they are quite robust.

Historically, the regulatory tests used in risk assessment examine one (or a limited number of) endpoint(s). Therefore, regulatory thresholds have typically been set using the dose at which a single, sensitive, apical endpoint is altered. However, unlike these standard assays, toxicogenomics tests tens of thousands of endpoints (including expression changes of individual genes, pathways, and ontologies); therefore, applying a traditional approach that favors a single, sensitive data-point and ignores the rest seems to defeat the purpose of performing a global analysis. Our third approach to PoD selection, selecting the mode, mean, or median of the BMD(L)-mean values (across all pathways), can also be applied to compounds with unknown MoAs (Fig 6C). However, we believe that using one of these metrics is a more robust approach to PoD selection because these values represent all of the data (as opposed to a single, sensitive data point). In particular, the mode represents the peak or maximum of the distribution, and therefore the dose at which the majority of the transcriptional responses occur, thereby ensuring that the PoD decision is anchored to a very large proportion of the transcriptional data. The distribution of the gene and pathway BMD(L) values for furan was unimodal (Fig 3), which facilitates the use of the mode (presumably, if a toxicant produced a multi-modal distribution, one would choose to use the mode of the most sensitive peak). The phenomenon that there is a unimodal peak in transcriptional activity that is reflected in the distribution of BMD values has also been reported for median BMD and BMDL values of naphthalene-dependent GeneGo signaling pathways [19]. Therefore, our third approach, selecting the mode, mean, or median of the BMD(L)-mean values (across all pathways), offers an alternative way to estimate the system’s perturbation that relies on the weight of evidence provided by the entire toxicogenomic dataset and allows for the selection of a PoD that reflects the BMD at which the majority of the transcriptional reaction to the compound occurs.

Unlike the first three approaches, our final method for PoD selection is appropriate for well-studied chemicals (such as furan). Using the BMD-mean of a MoA-relevant pathway requires at least some understanding of the chemical’s biological effects, which is an important aspect of establishing the human relevance of rodent data [36]. We previously argued that chronic activation of the Nrf2 transcription factor plays a pivotal role in the malignant transformation of regenerating liver tissue following furan-induced injury [3]. Therefore, we chose the *Nrf2-mediated Oxidative Stress Response pathway* as the MoA-relevant transcriptional endpoint for furan-induced liver cancer (Fig 6D). Interestingly, this approach was the best at predicting the dose at which the malignant form of furan-induced cancer (HCC) occurs. Therefore, using a MoA-specific BMD may be helpful aligning the BMD selected for PoD with the more severe adverse outcomes. In a recent study by Moffat et al. [1], the authors compared the results of three independent risk assessments of benzo[*a*]pyrene (BaP): one that used only apical data, one that used only transcriptomic data, and one that was a hybrid of the two. The transcriptional PoD for BaP was selected based on BMD modeling of MoA-relevant pathways and they reported that the conclusions made using these data were consistent with the conclusions that were made in the traditional and hybrid assessments. In another study, a correlation between MoA-relevant pathway BMD values and histological BMD values was reported in which tissue- and gender-specific BMD differences observed for histological endpoints were conserved in the transcriptional BMD modeling results [19]. Thus, deriving a PoD from a MoA-relevant key event is a suitable approach for well-characterized compounds.

Regulatory agencies worldwide are challenged with assessing health risks for legacy chemicals and products, many of which have minimal toxicity information. It has been proposed that toxicogenomics is an effective screening tool for such data-poor chemicals because toxicogenomics experiments can be conducted more quickly and using fewer resources than many standard approaches [37]. Work from our laboratory has demonstrated that toxicogenomics data are useful for both mechanistic and quantitative risk assessment. Here we show that BMD values generated for the most sensitive pathways, the transcriptional modes, means, and medians, or individual MoA-relevant pathways were highly consistent with one another, with furan-dependent cancer BMDs, and across genomics platforms. We note that statistical filtering of data prior to modeling creates BMDs that are more conservative and less likely to over-estimate the adverse-outcome that they are intended to predict. We caution that it is possible that the remarkably high concordance rates achieved here may be, at least partially, due to the limited dose range used. Therefore, we recommend that this type of study be repeated using additional chemicals and more extensive dose ranges. It seems clear that there are a number of approaches that can be effectively used to choose a PoD dose, and that most of these do not require prior knowledge of the compound’s toxicity. This study represents an important step toward confidently and effectively applying toxicogenomic data to quantitative risk assessment. The comparisons made here can be used to make evidence-based decisions regarding the experimental design of future toxicogenomics studies that include BMD modeling.

## Supporting Information

S1 Table RNA-Seq. Differentially expressed gene list.
(XLSX)

S2 Table Microarray. Differentially expressed gene list.
(XLSX)

S3 Table qPCR. Differentially expressed gene list.
(XLSX)

S4 Table Best dichotomous models for dose-response of number of DEGs for RNA-seq, microarray, and qPCR experiments in response to 0, 2, 4, 8 mkd furan. (XLSX)

S5 Table Statistics for box-and-whisker plots, Fig 2, which show that (within each genomics platform) means are significantly different. (XLSX)

S6 Table Percent gene or pathway BMDs within the apical BMD range for cancer (2.6–5.13 mkd for HCA-HCC) for each technology. (XLSX)

S1 Fig The intra-dose, inter-platform overlap of DEGs (by unique gene symbol; top) and pathways (bottom). (TIF)

S2 Fig Filtering gene expression data for statistically significant changes prior to modeling in BMDExpress significantly changes the distribution of pathway BMDL-mean values. Distributions of pathway BMD-mean values for RNA-seq (top), microarray (center) and qPCR (bottom). Mode values are labeled. Modes decrease as filtering stringency increases (unfiltered = navy blue, ANOVA filtered = light blue, FDR filtered = red). Pathways were only considered in this analysis if they had 4 or more molecules with p fit>0.1. Overlain are the number of transcripts used to model each group.
(TIF)

S3 Fig Model counts (pie charts) and gene BMD/BMDL plots (scatterplots) for each platform. Linear regressions comparing BMD/BMDL values were R2 > 0.9 (linear regression p < 0.0001), with a slopes of 0.66–0.75 (corresponding to a BMD/BMDL ratio of 1.5–1.3).
(TIF)

S4 Fig Inter-platform comparisons of BMDLs for genes (top), pathway-means (center), and pathway medians (bottom) for ANOVA filtered data. Statistically significant correlations are indicated in red (regression p<0.05).
(TIF)

S5 Fig Inter-platform comparisons of BMDs (left) and BMDLs (right) for genes (top), pathway-means (center), and pathway medians (bottom) for FDR filtered data. Statistically significant correlations are indicated in red (regression p<0.05).
(TIF)

S6 Fig Inter-platform comparisons of BMDs (left) and BMDLs (right) for genes (top), pathway-means (center), and pathway medians (bottom) for unfiltered data. Statistically significant correlations are indicated in red (regression p<0.05).
(TIF)
