# Supplementary figures and images for: Impact of Genomics Platform and Statistical Filtering on Transcriptional Benchmark Doses (BMD) and Multiple Approaches for Selection of Chemical Point of Departure (PoD)
Source: PLoS One. 2015 Aug 27;10(8):e0136764. doi: 10.1371/journal.pone.0136764 (PMC4551741; doi:10.1371/journal.pone.0136764)

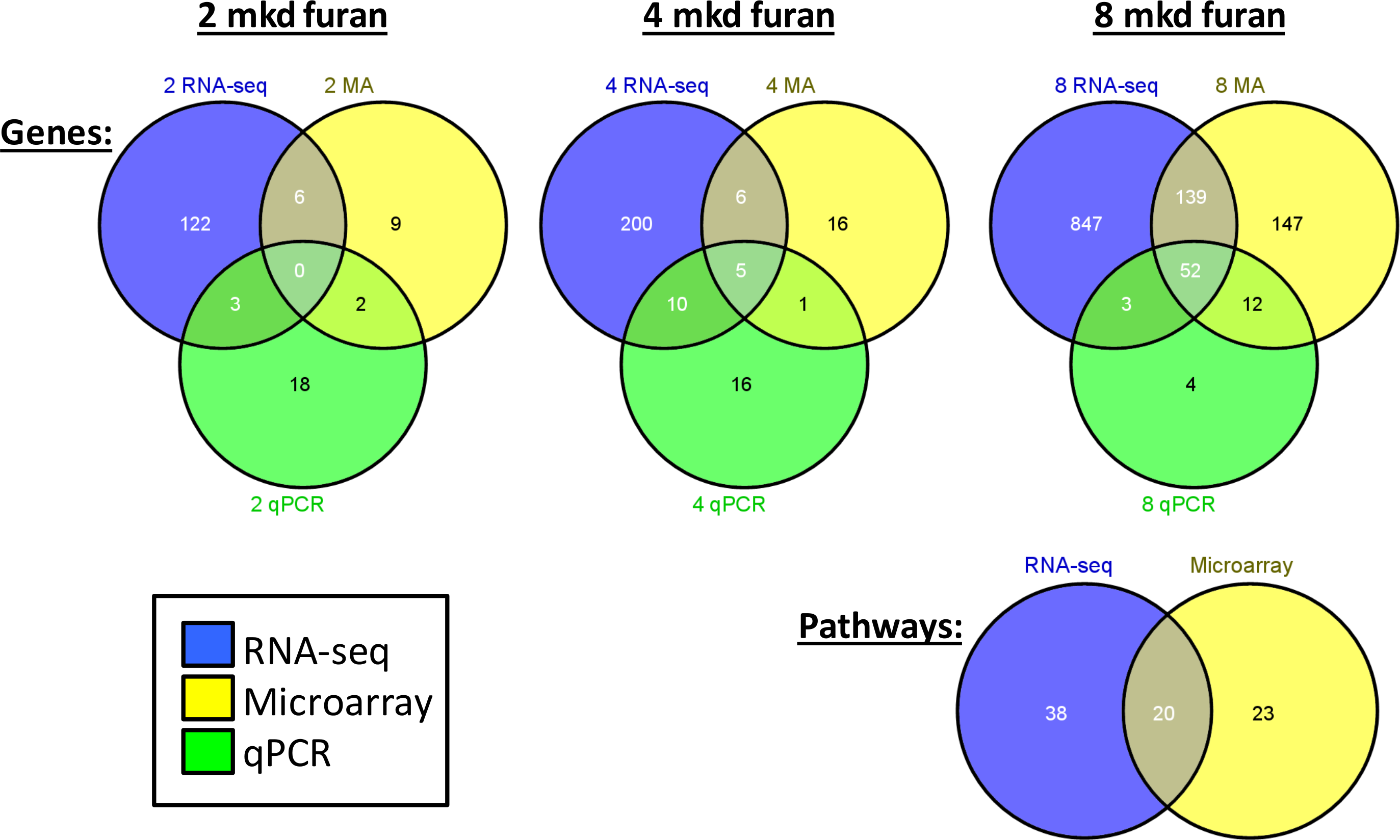

Supplement: S1 Fig — (TIF) [file pone.0136764.s007.tif]

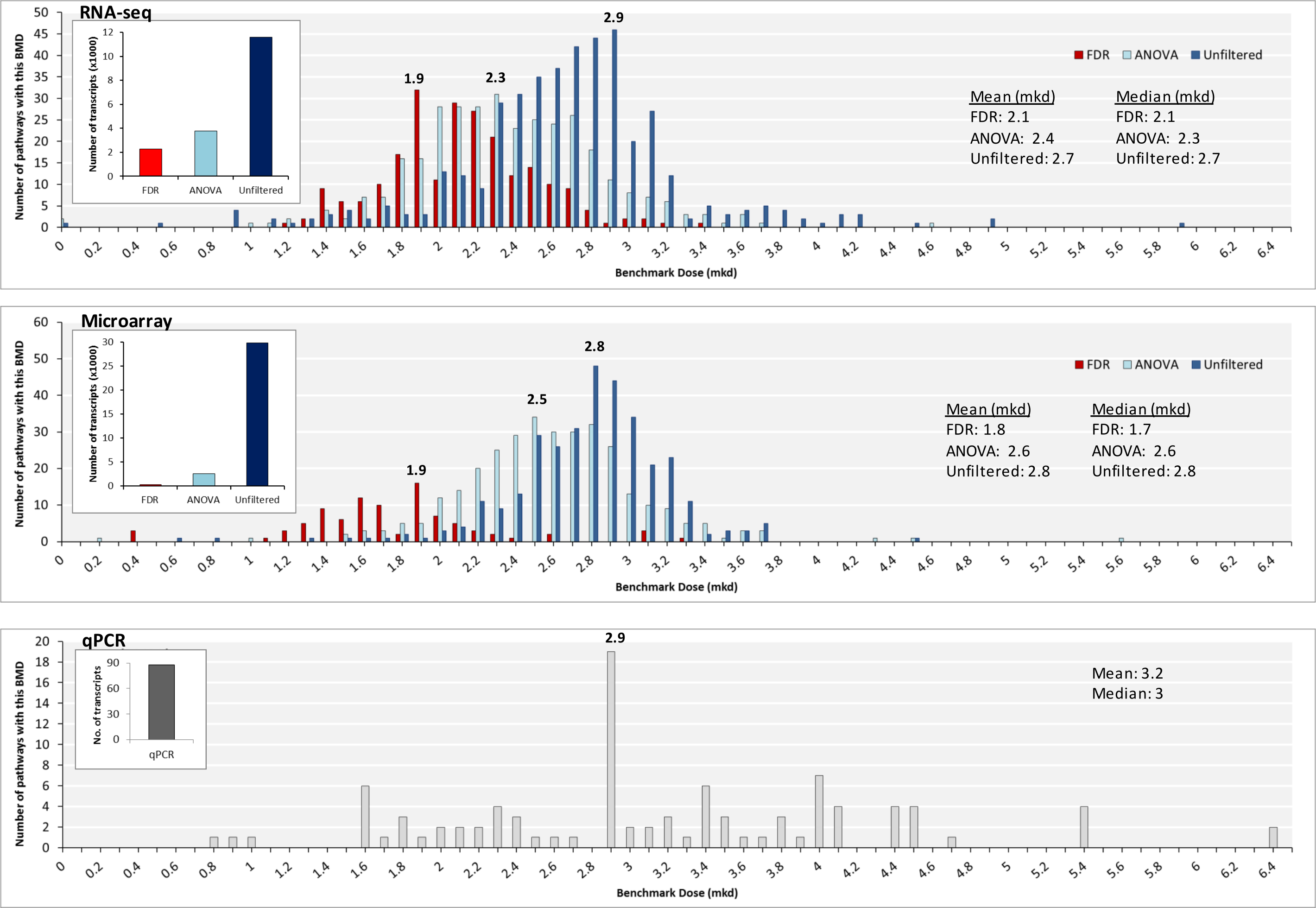

Supplement: S2 Fig — Distributions of pathway BMD-mean values for RNA-seq (top), microarray (center) and qPCR (bottom). Mode values are labeled. Modes decrease as filtering stringency increases (unfiltered = navy blue, ANOVA filtered = light blue, FDR filtered = red). Pathways were only considered in this analysis if they had 4 or more molecules with p fit>0.1. Overlain are the number of transcripts used to model each group. (TIF) [file pone.0136764.s008.tif]

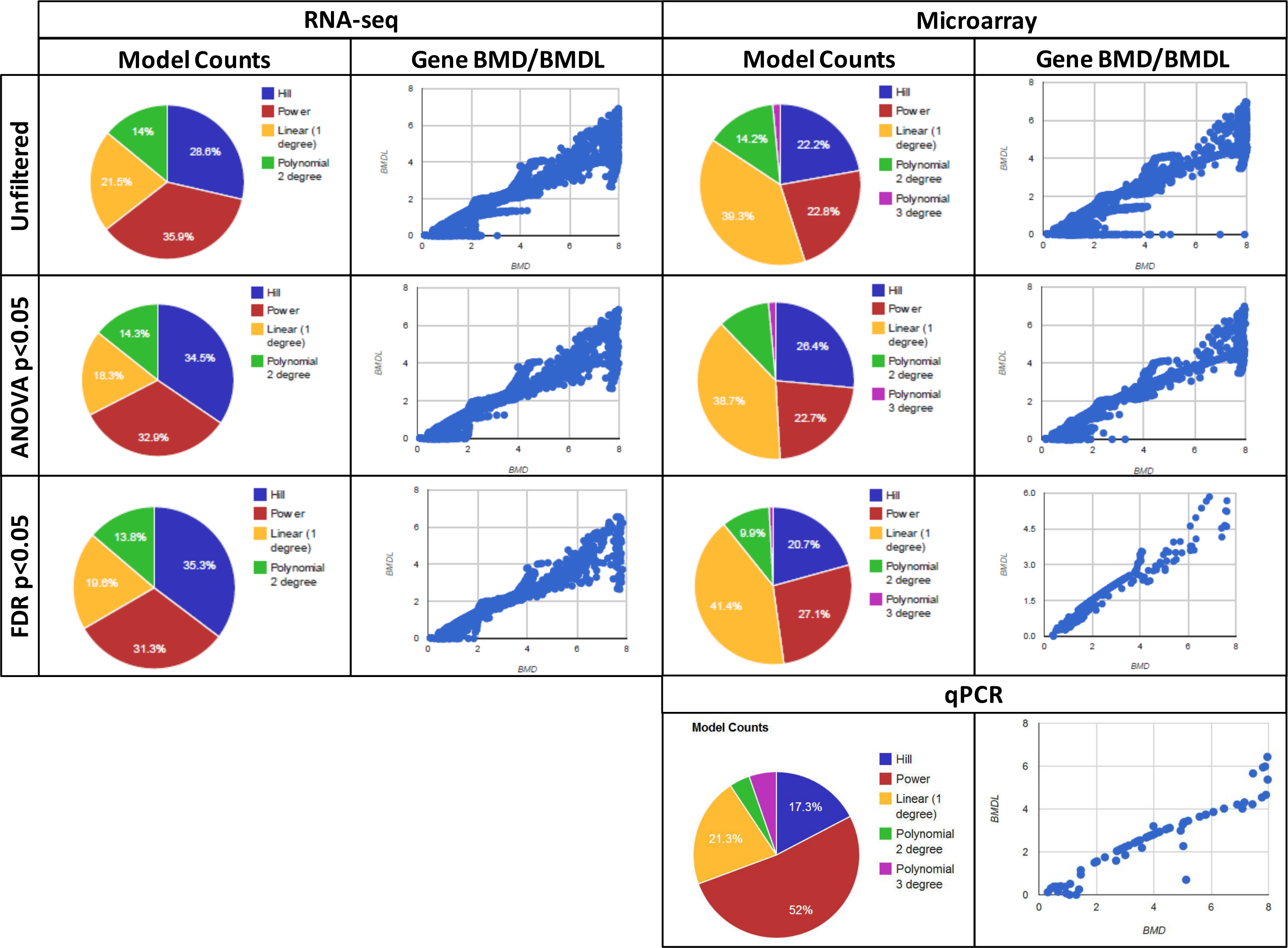

Supplement: S3 Fig — Linear regressions comparing BMD/BMDL values were R2 > 0.9 (linear regression p < 0.0001), with a slopes of 0.66–0.75 (corresponding to a BMD/BMDL ratio of 1.5–1.3). (TIF) [file pone.0136764.s009.tif]

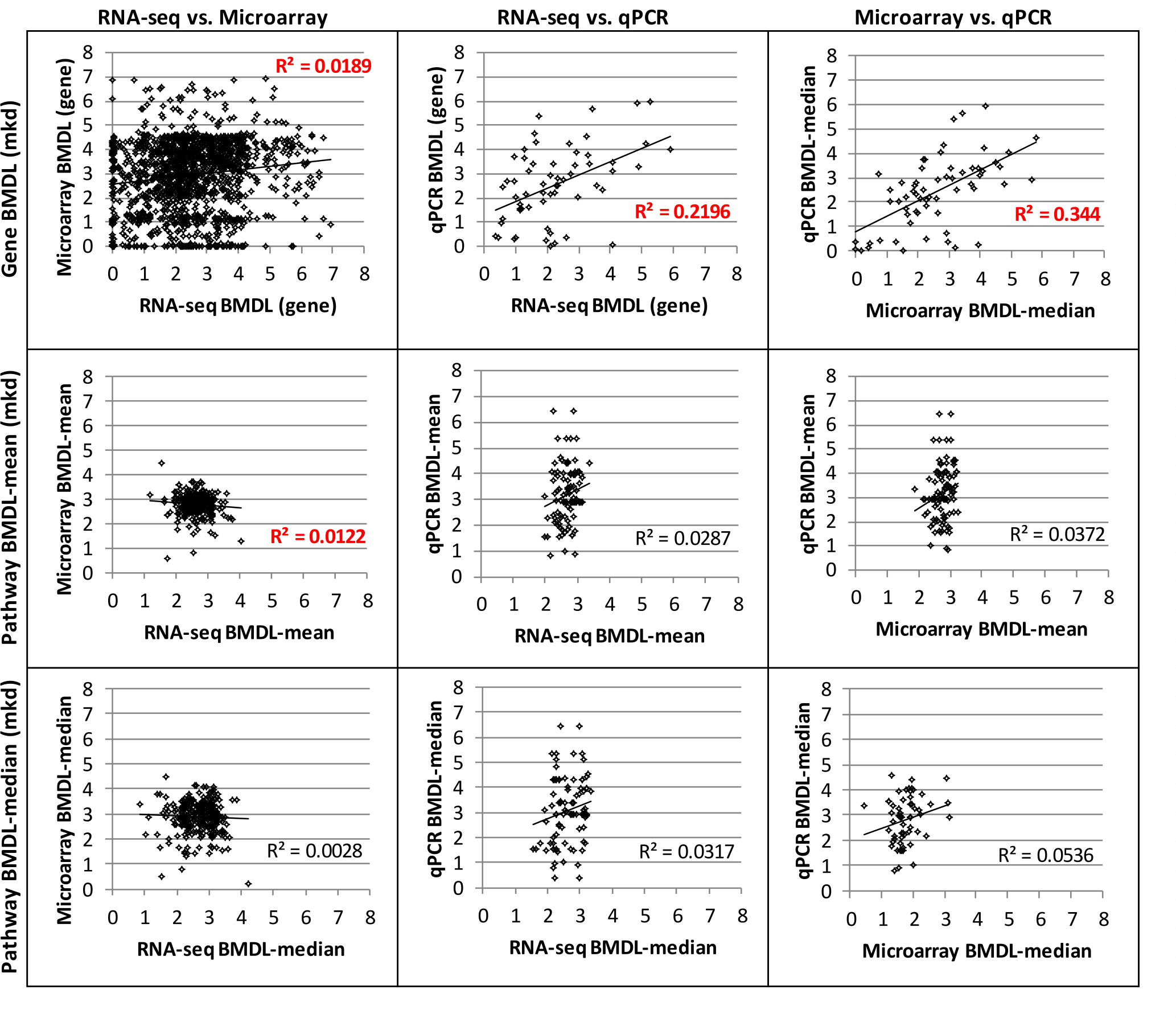

Supplement: S4 Fig — Statistically significant correlations are indicated in red (regression p<0.05). (TIF) [file pone.0136764.s010.tif]

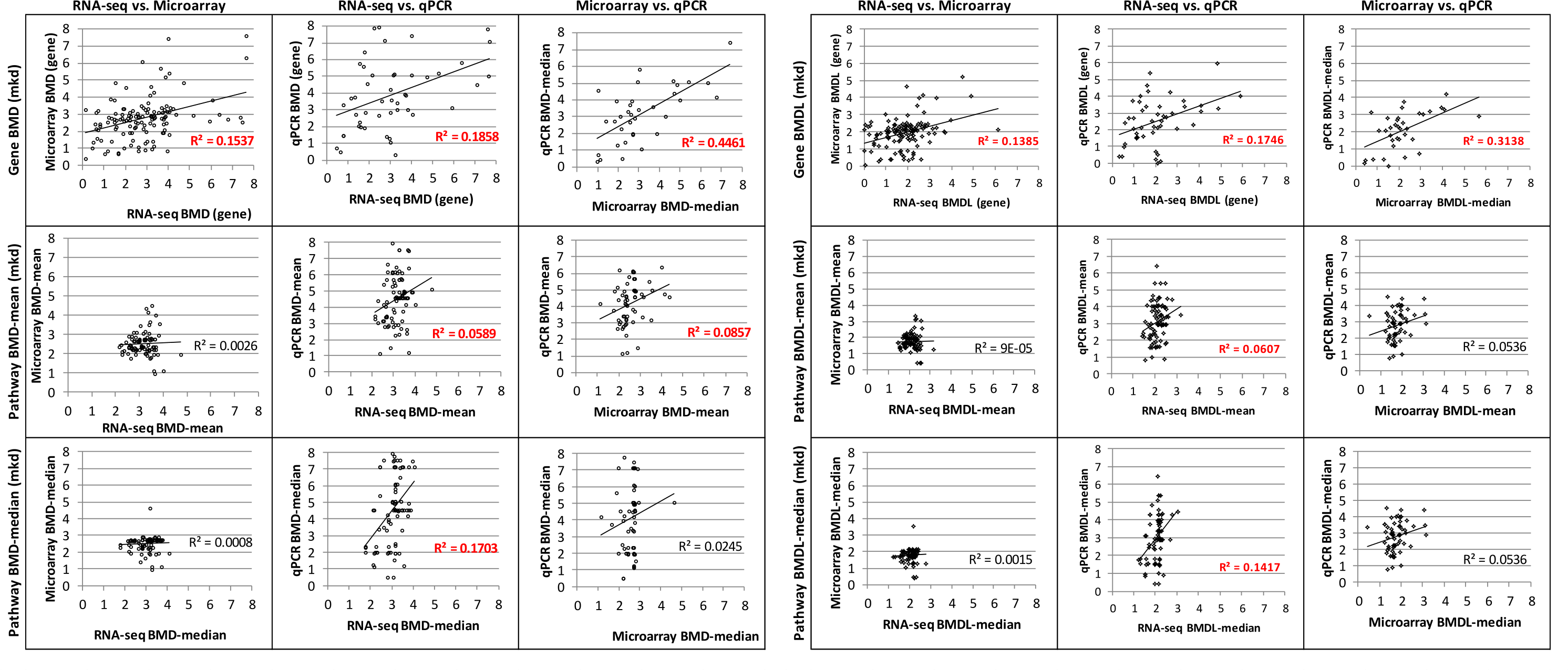

Supplement: S5 Fig — Statistically significant correlations are indicated in red (regression p<0.05). (TIF) [file pone.0136764.s011.tif]

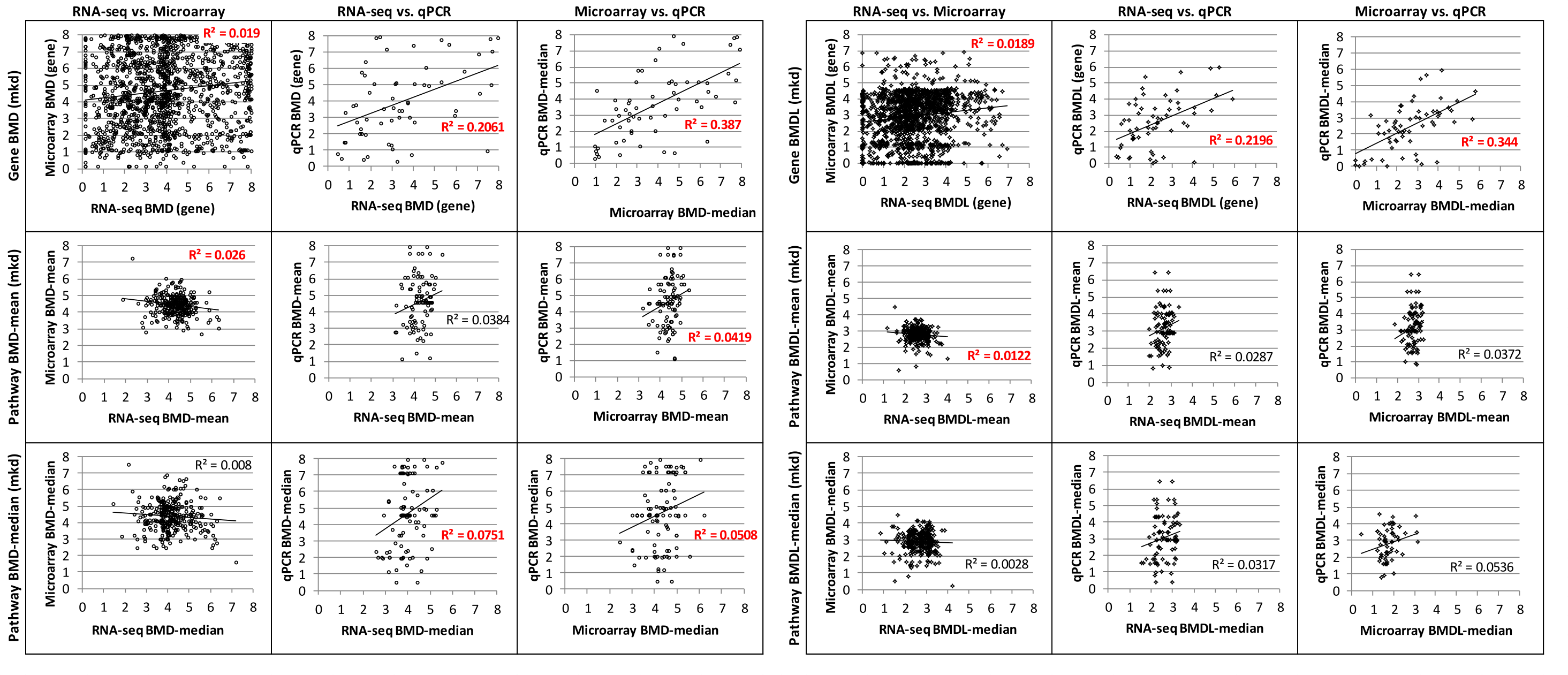

Supplement: S6 Fig — Statistically significant correlations are indicated in red (regression p<0.05). (TIF) [file pone.0136764.s012.tif]
